# Using bundle embeddings to predict daily cortisol levels in human subjects

**DOI:** 10.1186/s12874-018-0485-y

**Published:** 2018-03-21

**Authors:** Roelof B. Toonen, Klaas J. Wardenaar, Elisabeth H. Bos, Sonja L. van Ockenburg, Peter de Jonge

**Affiliations:** 1 Department of Developmental Psychology, Heymans Institute for Psychological Research, University of Groningen, Faculty of Behavioural and Social Sciences, Grote Kruisstraat 2/1, 9712 TS Groningen, the Netherlands; 2 Interdisciplinary Center Psychopathology and Emotion Regulation (ICPE), University of Groningen, University Medical Center Groningen, CC72, Hanzeplein 1, 9700 RB Groningen, the Netherlands

**Keywords:** Time series, Nonlinear dynamic systems, Cortisol, Bundle embeddings, Prediction

## Abstract

**Background:**

Many biological variables sampled from human subjects show a diurnal pattern, which poses special demands on the techniques used to analyze such data. Furthermore, most biological variables belong to nonlinear dynamical systems, which may make linear statistical techniques less suitable to analyze their dynamics. The current study investigates the usefulness of two analysis techniques based on nonlinear lagged vector embeddings: sequentially weighted global linear maps (SMAP), and bundle embeddings.

**Methods:**

Time series of urinary cortisol were collected in 10 participants, in the morning (‘night’ measurement) and the evening (‘day’ measurement), resulting in 126 consecutive measurements. These time series were used to create lagged vector embeddings, which were split into ‘night’ and ‘day’ bundle embeddings. In addition, embeddings were created based on time series that were corrected for the average time-of-day (TOD) values. SMAP was used to predict future values of cortisol in these embeddings. Global (linear) and local (non-linear) predictions were compared for each embedding. Bootstrapping was used to obtain confidence intervals for the model parameters and the prediction error.

**Results:**

The best cortisol predictions were found for the night bundle embeddings, followed by the full embeddings and the time-of-day corrected embeddings. The poorest predictions were found for the day bundle embeddings. The night bundle embeddings, the full embeddings and the TOD-corrected embeddings all showed low dimensions, indicating the absence of dynamical processes spanning more than one day. The dimensions of the day bundles were higher, indicating the presence of processes spanning more than one day, or a higher amount of noise. In the full embeddings, local models gave the best predictions, whereas in the bundles the best predictions were obtained from global models, indicating potential nonlinearity in the former but not the latter.

**Conclusions:**

Using a bundling approach on time series of cortisol may reveal differences between the predictions of night and day cortisol that are difficult to find with conventional time-series methods. Combination of this approach with SMAP may especially be useful when analyzing time-series data with periodic components.

## Background

Many biological variables that are sampled from human subjects show a diurnal pattern, which may reflect a rhythm innate to the responsible biological system or synchronization of the measured marker with the internal biological clock [1]. For example, human cortisol is known to increase in the morning – the so-called morning awakening response [2]-, followed by a decay over the rest of the day. When using conventional time-series analysis techniques to predict future values of such a variable, for example by fitting an autoregressive (AR) model, complications can arise due to the presence of the diurnal patterns. The estimated predictive performance of an AR model would by default be overestimated, because the fixed diurnal pattern renders the prediction error much smaller than the variance within the data. To adjust for effects of diurnal patterns, a common approach is to subtract the average time-of-day (TOD) value from the observed TOD value, either by including dummy variables for the TOD in the linear AR model or by using the residuals from the detrended series [3]. However, this approach may have some disadvantages when the studied variable belongs to a nonlinear dynamical system, which is the case for many biological variables. First, detrending time series by either subtracting a general linear trend or by subtracting the average TOD value is not necessarily allowed for nonlinear time series, because the data cannot be described as the linear sum of the values of independent processes [4]. Second, many biological dynamical systems are forced by external diurnal variables. When a nonlinear dynamical system is forced by a periodically oscillating variable, the dynamics of the forced system may depend upon the phase of the forcing variable [5]. Applying a linear AR model (such as vector autoregressive [VAR] models) to the system’s variables would not take into account the possibility of having different relationships between variables at different TOD values. Linear models yield single coefficients that do not change over time. Adding extra factors to account for such differences in the relationships at different time points is not suitable for nonlinear systems, because it is impossible to add independent linear contributions. To overcome the abovementioned disadvantages, the current study was aimed to investigate an alternative approach, based on the theory of periodically forced nonlinear dynamical systems and lagged vector embeddings.

Central to the lagged-vector-embedding approach is the representation of a system’s dynamics by a trajectory through the system’s so-called *phase space*. Each dimension in a phase space corresponds to one of the relevant variables of the system. Each point in the phase space therefore corresponds to a different combination of the variables’ values. An essential difference between phase spaces and a classical time-series representation of the progression of a system’s dynamics through time is that a time-series representation – either univariate or multivariate – always has a ‘time’ axis. However, in a phase-space representation, a ‘time’ axis is not included. Instead, time is implicitly included as follows: each point in the phase space corresponds to a different moment in time and by moving from point to point along the phase-space path, the progression through time of the system can be traced.

**Fig. 1:**
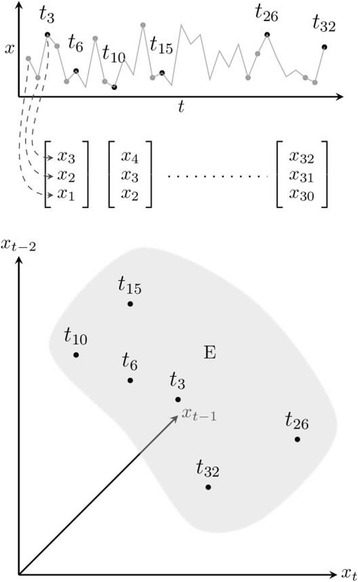
Construction of an embedding. In this example, with e = 3 and τ = 1, three-dimensional coordinate vectors are produced from time series x(t). The scalar components of each vector are obtained by taking lagged values of x at time t, t-1 and t-2. The embedding E is the set of all generated coordinate vectors. A dynamical path, connecting subsequent vectors in the embedding, has been omitted for the sake of clarity

If time-series data is available for all relevant variables, the phase-space trajectory can be constructed by taking the value of each variable at a specific moment in time and use these as the coordinates of a point in the phase space. Then repeat this for subsequent moments in time and connect the points. However, biological time-series data may not be available for all relevant variables of the system. Fortunately, according to the Takens theorem, the time-series data of a single variable often contain information about the complete system [6]. This means that the dynamical path can be reconstructed by constructing so-called lagged vector embeddings. If time-series data of an observed variable x are represented by (x_1_, x_2_,...,x_N_), where N is the number of observations, then an e-dimensional lagged vector *r*, with lags of τ, at time t, can be represented as: r_t_ = (x_t_, x_t-τ_, x_t-2τ_,..., x_t-(e-1)τ_) (see Fig. 1). At the optimal values of *e* and *τ*, the path consisting of all points r_t_ resembles the path in the space of all variables of the system. To determine the optimal parameters e and τ, several methods are available [7]. However, these methods may not be optimal in the presence of noise. In that case, it may be necessary to try a range of parameter values.

In the case of periodically forced dynamical systems, embedding construction is not as straightforward as described above [5]. Instead of constructing a single embedding, consisting of all vectors, the lagged vectors are grouped according to the TOD value of the first scalar component x_t_ in r_t_. Each group of vectors forms a separate embedding on its own, the so-called *bundle embedding*. This way, the original set of vectors r_t_ = (x_t_, x_t-τ_, x_t-2τ_,..., x_t-(e-1)τ_) is now split into M subsets r^m^_t_ = (x_t_, x_t-τ_, x_t-2τ_,..., x_t-(e-1)τ_), where M is the number of measurements per day and m is the TOD label (with m = t modulo M). Any analysis technique that is suitable for use with normal embeddings can also be applied to these bundle embeddings. In this way, periodically forced dynamical systems can be analyzed without having to apply linear techniques to correct for the periodicity (e.g. diurnal rhythms) in the values of its variables.

To predict future values in time-series data by means of embeddings, the sequentially weighted global linear map (SMAP [8]) is an elegant and flexible technique because it also provides information about the amount of nonlinearity that may be present within the time series. Furthermore, SMAP is a nonparametric technique, meaning that no a priori assumptions need to be made about the underlying nonlinear model. Although nonlinear systems are governed by nonlinear mathematical relationships, it is often possible to fit linear mathematical models locally to an embedding. This means that at each point in the embedding a linear model may correctly describe the behavior of the system in a small neighborhood of that point. However, the parameter values of such a linear model differ from the parameter values at another position in the embedding. To fit a linear model to a particular neighborhood, only the vectors from that neighborhood would be used. Ideally, that neighborhood would be small and only very few vectors would be needed. However, in the case of noisy data, using more vectors (that is: increasing the size of the neighborhood) may give more accurate estimations. In the case of purely linear systems, the parameter values of each local linear model would theoretically be the same. In those cases it would be better to fit only one model and use all vectors to estimate the model parameters. The SMAP results would then be comparable to results obtained by using a standard linear technique (for example: vector autoregression) on the complete time series. The SMAP method provides a flexible way of selecting the size of the neighborhood by using a Gaussian weight function on the embedding’s vectors. The width of the optimal weight function provides extra information about the type of system that is being studied. Small weight functions suggest strong local behavior, which may be an indication of a nonlinear underlying system. Broad weight functions may indicate a purely linear system or the presence of large amounts of noise.

**Fig. 2:**
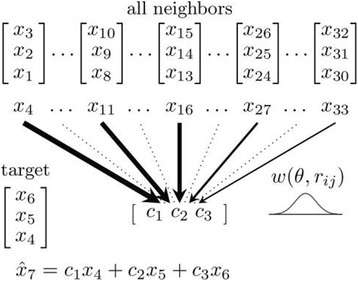
The SMAP procedure. The SMAP procedure is illustrated for the target vector at *t* = 6 and for e = 3. The other vectors in E are now regarded as neighbors of this target. Each neighbor has an associated future value of x at time t + 1. The set of neighbors and their associated values is used to construct a local linear model with 3 parameters (c_1_, c_2_, c_3_). These parameters only apply to a neighborhood of the target vector. When constructing this linear model, each neighbor is assigned a weight depending upon its distance to the target in embedding E, using a Gaussian function w. The width of w depends upon parameter θ. The target’s future value (x_7_) is computed using the linear model with scalar values from the target vector. Thus, a separate model and prediction are generated for each vector in E. The prediction performance is computed by comparing the predicted values with the observed values

Given an e-dimensional embedding E, SMAP predicts the future time-series value x_t + 1_ by using the future values of all neighbor vectors r_i_ of the target vector r_t_ = [x_t_, x_t-1_,...x_t-(e-1)_]in the embedding (see Fig. 2). It does so by fitting a linear model x_t + 1_ = c_1_x_t_ + c_2_x_t-1_ + … + c_e_x_t-(e-1)_, to the neighbor vectors and their future values, using a total least squares procedure. When estimating the linear model, vectors close to the target r_t_ are assigned a greater weight than distant ones. These weights are assigned on the basis of the Gaussian function w(d) = exp(−θd/d_avg_), where d is the Euclidian distance to the target vector within the embedding space, d_avg_ is the average Euclidian distance between vectors, and θ controls the width of the function. A θ value equal to zero results in a function of infinite width, and equal weights are assigned to each vector. This corresponds to the ‘global linear case’, and the fitted model is comparable to a standard VAR model [9]. For values of θ greater than zero, the fitted linear model becomes more local, suggesting there is more nonlinearity in the underlying system. For large values of θ the weight approaches zero rapidly, effectively limiting the neighborhood to the closest vectors only.

The current study aimed to investigate the usefulness of a combination of SMAP and bundle embeddings in the analysis of biological time-series data that are known to show a diurnal pattern. To this end, analyses were conducted in urinary cortisol time-series data from 10 adult participants, who collected two batches per day of their accumulated urine, during a period of 63 consecutive days. For each of the resulting cortisol time series, SMAP model predictions were estimated and prediction accuracy was compared between models with full embeddings (i.e. unbundled), bundled embeddings, and embeddings based on TOD-corrected time-series data. These analyses were conducted using a range of embedding dimensions and values of the width parameter. A bootstrap procedure was used to estimate prediction standard errors to gain insight into estimation precision.

## Methods

### Participants

Urinary cortisol data were obtained from 10 participants (7 women and 3 men), with ages ranging from 19 to 58 years, as part of a daily diary study, with a duration of 63 days [10]. Participants were recruited by poster adverts in the city of Groningen, the Netherlands. Excluded were subjects using medication other than oral contraceptives or occasional acetominophen, and subjects with a current somatic or mental illness. The study was approved by the Medical Ethics Committee of the University Medical Center Groningen. The participants provided informed consent before enrollment.

### Urinary cortisol

For each participant, two containers of urine were collected per day. The ‘night’ container contained all urine that was produced after the participant went to bed and included the first morning void after awakening. The ‘day’ container was used to collect all urine that was produced during the day, up to and including the last void before going to bed. Containers were collected every three days. Until that time, the containers were stored at room temperature at the participants home. After collection, samples were taken using 2 mL cups. These were stored at a temperature of − 80 °C. After 63 consecutive days, cortisol levels in all 126 samples of a single participant were determined in one run, using liquid chromatography tandem mass spectrometry [11]. Lower range intrarun coefficients of variation were 2.4%. The higher range intrarun coefficients were 1.4%.

### Embedding construction and bootstrapping

Before construction of the full embeddings and the bundle embeddings, outliers having a deviation greater than 2.5 standard deviations (SD) from the mean were removed from the cortisol time series (see Table 1). After this, first differences were taken to remove any long-term linear trends [12], and the resulting time series were standardized to zero mean and unit standard deviation for each individual separately. Embeddings with dimension e and lag size τ were constructed by extracting e-dimensional coordinate-vectors from these first-differenced standardized cortisol time series (x), and by combining the points corresponding to these vectors. To obtain a vector at time t, the values of (x_t_, x_t-τ_, x_t-2τ_,...,x_t-[e-1]τ_) were taken from x.

**Table 1:** Summary statistics for cortisol

	Night			Day		
ID	n	mean	sd	n	mean	sd
1	60	23.8	11.9	58	46.7	18.8
2	61	15.2	3.7	61	17.1	4.8
3	61	10.3	6.2	61	50.6	15.2
4	62	30.3	14.1	61	61.6	23.7
5	62	18.1	9.0	62	99.8	38.5
6	62	31.6	19.4	60	78.3	23.3
7	60	17.5	12.7	61	80.3	29.7
8	61	26.8	19.5	60	68.4	32.0
9	60	20.2	18.1	63	58.7	25.6
10	59	23.4	12.7	59	79.1	24.5

Values were obtained after removal of outliers, but before first-differencing and standardizing. Note: ID = subject ID, n = number of data points in the time series, mean = mean value (nmol), sd = standard deviation (nmol)

For the current analysis, the lag value τ was limited to 1. Using greater lag sizes would result in vectors spanning a broader part of the time series, and would therefore decrease the number of available vectors, which was not desirable given the relatively short length of the time series.

For each participant, nine full embeddings, with dimensions ranging from 1 to 9, were extracted from the time-series data and prediction accuracy was evaluated for each resulting model. For each embedding, 5000 bootstrap embeddings were created by picking vectors randomly from the original embedding, while keeping the number of vectors per bootstrap embedding the same as in the original embedding. The relative occurrence of ‘day’ and ‘night’ vectors – where a ‘day’ or ‘night’ vector is a vector where the first scalar value, x_t_*,* is a ‘day’ or ‘night’ value respectively – was kept the same as in the original embedding. To obtain the bundle embeddings, the full embeddings were split into two bundles each: the ‘day’ bundle, containing only ‘day’ vectors, and the ‘night’ bundle, containing only ‘night’ vectors.

To obtain the TOD-corrected embeddings, the mean day and night cortisol values were calculated and subtracted from the day and night values respectively (after removal of the outliers). To facilitate comparison of the results for the different embedding types, the resulting time series were first-differenced and standardized. After this, embeddings were constructed in the same way as for the full embeddings, using the same bootstrap procedure.

### Local linear model prediction

When fitting the local linear SMAP models, a total least squares (TLS) procedure was used to compute the regression coefficients [13]. When computing the TLS coefficients, the target vector itself was excluded from the set of available neighbor vectors, effectively making this a leave-one-out cross-validation procedure. Parameter θ, which controls the width of the weight function, was varied between 0 (producing a global linear model) and 3 (producing a strongly local linear model), with incremental steps of 0.2. The prediction accuracy of the fitted model was computed at each value of θ and for each bootstrap embedding and each day or night bundle. In order to do this, the normalized root mean square error (NRMSE) of the predicted values relative to the observed values was computed. The NRMSE was obtained by dividing the root mean square error (RMSE) by the SD of the time series. An NRMSE smaller than one indicates a better than chance performance of the fitted model, while an NRMSE greater than one indicates a worse than chance performance. Because the time series of different participants showed different standard deviations, a comparison of model fit across participants, based solely on the (non-normalized) RMSE, would be less informative.

To compute a model for a target in a specific bundle, only vectors from the same bundle were used. The bootstrap distribution of the regression coefficients was used to estimate confidence intervals (CI) for these coefficients.

Finally, the prediction accuracy of the different types of embedding (full, bundled, TOD-corrected) were compared, by means of a Mann-Whitney U test on the distributions of the computed NRMSEs.

## Results

### Guide to reading the tables and figures

**Fig. 3:**
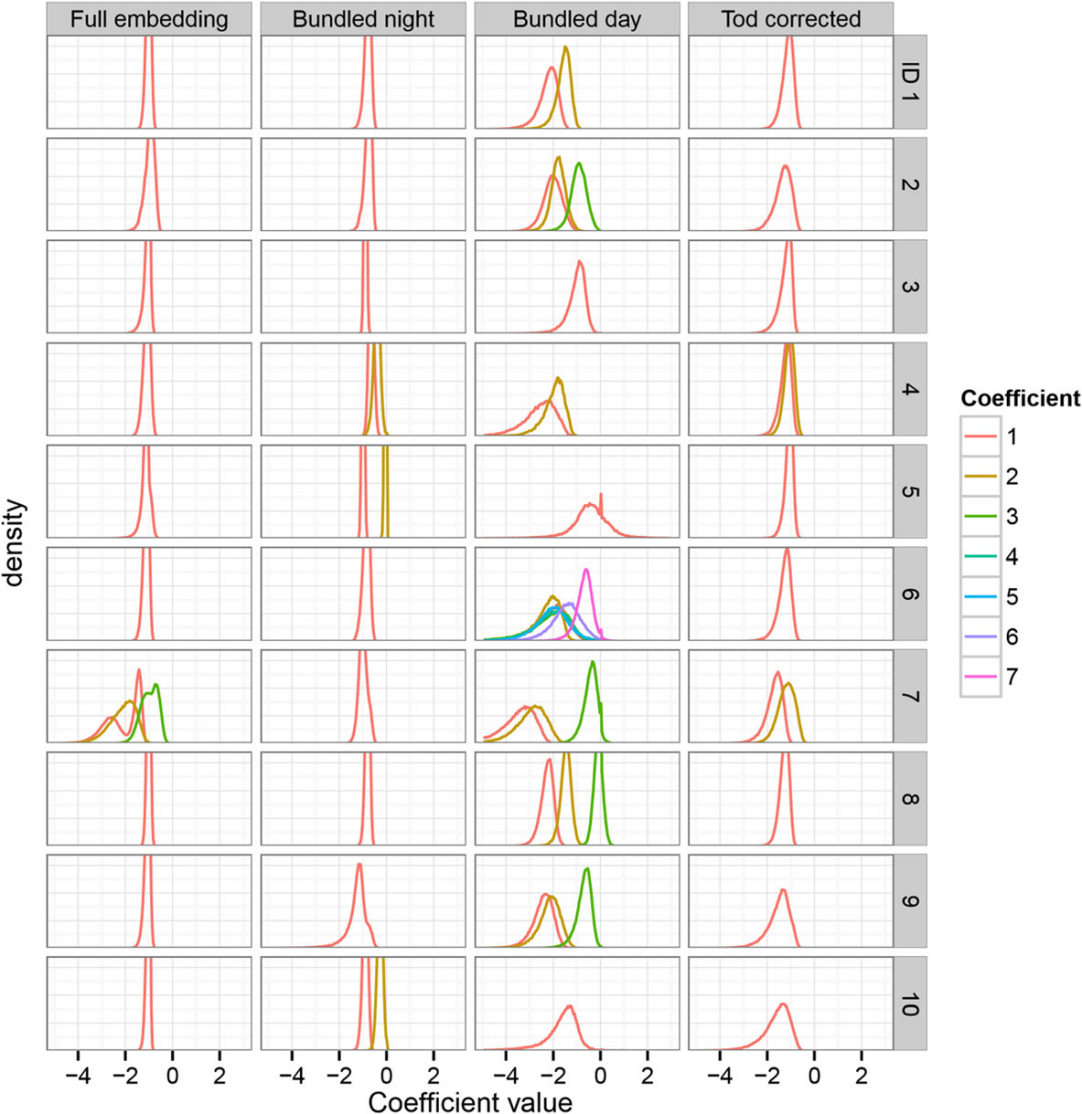
Coefficient overall distributions for the optimal embeddings. The overall distributions of the coefficients of the fitted (local) linear models are shown for the optimal embeddings of the three different analyses (full, bundled, TOD-corrected) and for each subject (ID). The densities of narrow distributions have been cut off at a value of 0.07. The number of coefficients (1 to 7) per embedding depended upon the number of embedding dimensions. The distributions of the intercepts (coefficient 0) were omitted from the plots because they all showed a high and narrow peak around 0

**Fig. 4:**
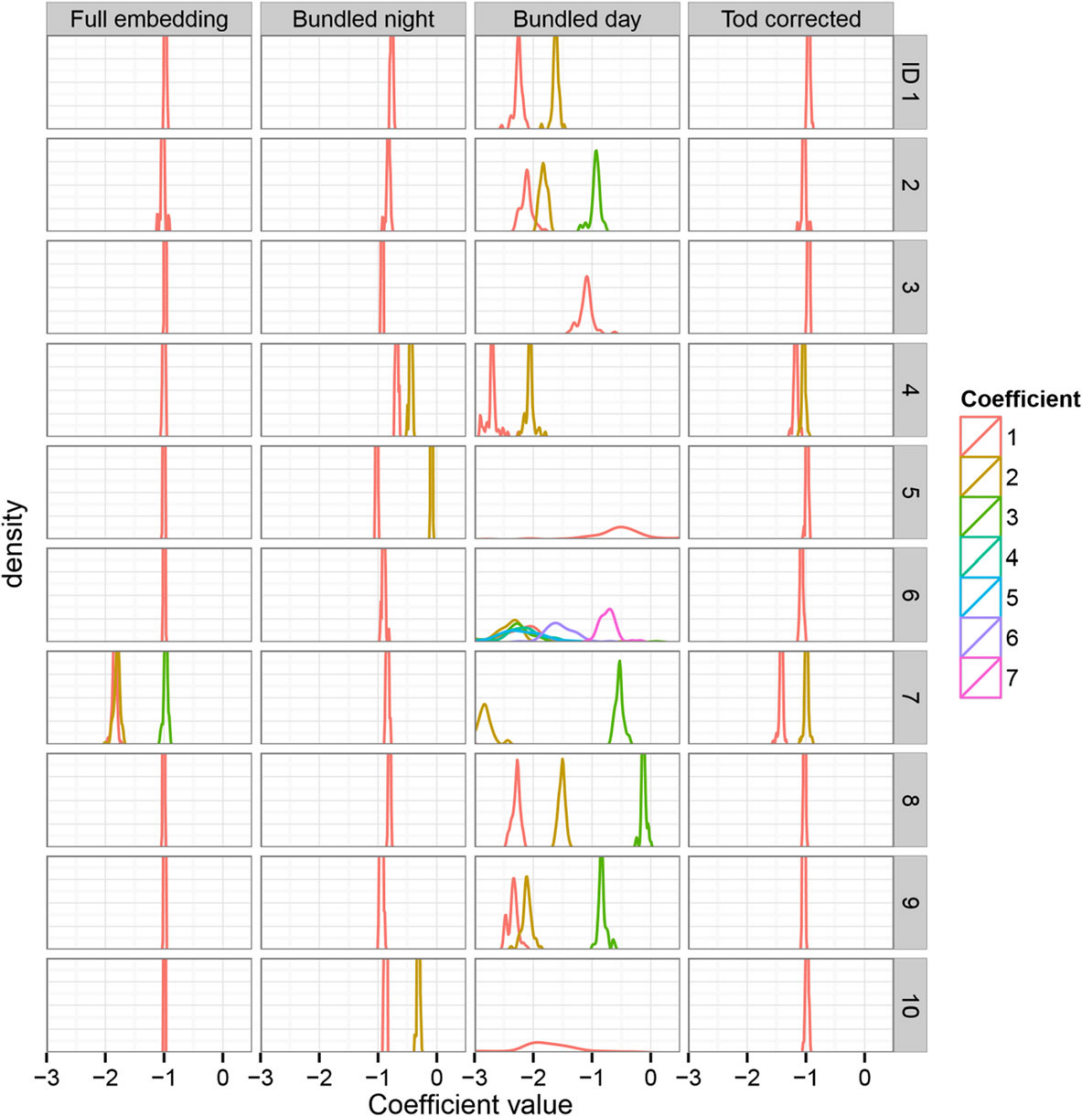
Coefficient intra-embedding distributions for the global linear models. For each target vector, the average of the bootstrap distribution of the coefficients of the corresponding global linear model (θ = 0) was computed. The distributions of these averages are shown for the three different analyses (full, bundled, TOD-corrected), for each subject (ID) and for each model coefficient. The distributions of the intercepts (coefficient 0) were omitted from the plots because they all showed a high and narrow peak around 0

**Fig. 5:**
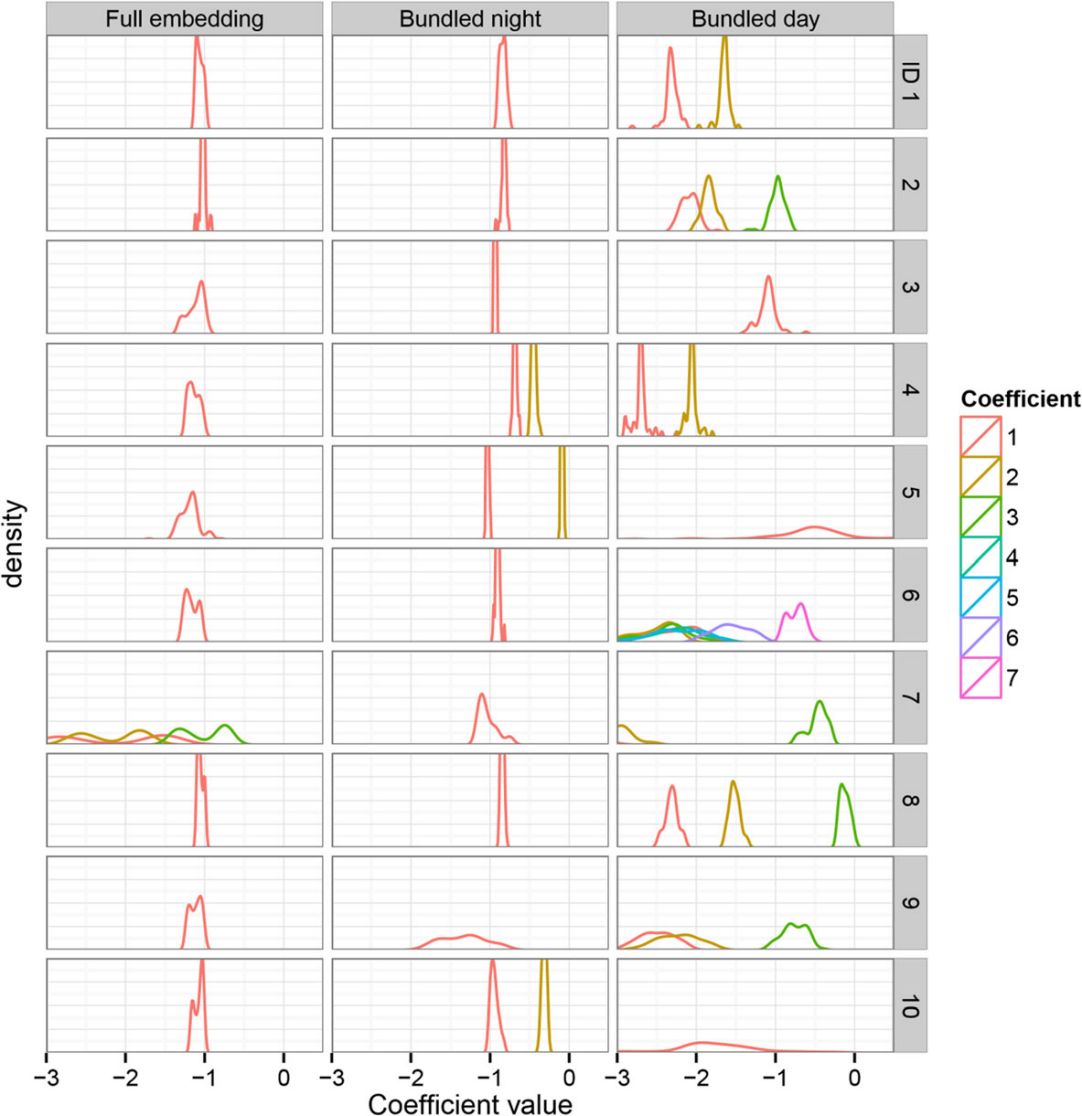
Coefficient intra-embedding distributions for the optimal models. For each target vector, the average of the bootstrap distribution of the coefficients of the corresponding optimal linear model (θ >= 0) was computed. The distributions of these averages are shown for two different analyses (full, bundled), for each subject (ID) and for each model coefficient. The distributions of the intercepts (coefficient 0) were omitted from the plots because they all showed a high and narrow peak around 0

Based on the NRMSE of the predictions, an optimal embedding was selected for each participant and for each analysis (full, Table 2; bundled, Table 3; TOD corrected, Table 4). Information about the precision and variation of the computed coefficients of the linear models was obtained from their distribution functions. These are shown in Fig. 3. The zeroth coefficient (the intercept) of each model, which corresponds to the average value of the time series, peaked sharply around zero in all analyses, due to the standardization that was carried out beforehand. Therefore, it has been omitted from the figures and tables. An interpretation of the distributions of the remaining coefficients is not straightforward, since the coefficients may vary *within* an embedding, due to local linearity, and *between* bootstrap embeddings, due to the influence of noise and influential points. An approximation of the shape of the first coefficient’s distribution *within* the embeddings was obtained by computing the average value over the bootstrap embeddings for each target’s first coefficient. The distribution of these averages is shown in Fig. 4 for the global models, and Fig. 5 for the optimal models. Table 5 contains an overview of the statistical properties of these distributions. The width of these distributions reflects the variation of this coefficient within the embedding, where global models are expected to show sharp distributions, and local models to show wider distributions. For the *between*-embedding distributions, an approximation of the first coefficient’s distribution shape was obtained by aligning the centers of the bootstrap distributions per target and taking averages over all targets, thereby removing the intra-embedding variation. Table 6 contains an overview of the corresponding statistical properties. An estimate for the width of these distributions was obtained by computing the root mean square difference (RMSD) of all values relative to the average value of their respective targets. These distributions contain information about the precision of the computed coefficients. The results for each analysis are described in the next sections.

**Table 2:** SMAP results for the full embedding

ID	dim	n	nrmse_min_	∆nrmse	θ
1	1	107	0.689	0.001	0.2
2	1	114	1.015	0.000	0.0
3	1	112	0.323	0.021^b^	1.0
4	1	115	0.762	0.004^b^	0.2
5	1	119	0.385	0.045^b^	1.0
6	1	112	0.538	0.014^b^	0.6
7	3	101	0.426	0.080^b^	0.8
8	1	111	0.560	0.002^a^	0.2
9	1	116	0.584	0.007^b^	0.4
10	1	110	0.410	0.006^b^	0.6

Note: ID = subject ID, dim = dimension of the embedding, n = number of vectors in the embedding, nrmse_min_ = minimum of the NRMSE, Δnrmse = difference between the minimal NRMSE (θ > = 0) and the NRMSE at θ = 0 (the global linear case), θ = value of θ where the NRMSE was minimal

^a^Significant at the 0.05 level

^b^Significant at the 0.01 level

**Table 3:** SMAP results for the bundled embeddings

	Night bundle					Day bundle				
ID	dim	n	nrmse_min_	∆nrmse	θ	dim	n	nrmse_min_	∆nrmse	θ
1	1	54	0.799	0.001	0.2	2	52	1.254	0.009^a^	0.2
2	1	57	0.877	0.000	0.0	3	53	1.246	0.005	0.2
3	1	56	0.454	0.002	0.2	1	56	1.505	0.000	0.0
4	2	55	0.802	0.006^a^	0.2	2	56	1.455	0.000	0.0
5	2	58	0.329	0.001	0.2	1	59	2.353	0.000	0.0
6	1	57	0.842	0.000	0.0	7	40	1.620	0.004	0.2
7	1	55	0.578	0.024^b^	0.6	3	51	1.307	0.033	0.4
8	1	56	0.680	0.001	0.2	3	52	0.744	0.005^b^	0.4
9	1	57	0.722	0.046^b^	0.6	3	56	1.136	0.069^b^	0.6
10	2	53	0.625	0.006^b^	0.4	1	55	2.292	0.000	0.0

Note: ID = subject ID, dim = dimension of the embedding, n = number of vectors in the embedding, nrmse_min_ = minimum of the NRMSE, *Δnrmse* difference between the minimal NRMSE (θ > = 0) and the NRMSE at θ = 0 (the global linear case), θ = value of θ where the NRMSE was minimal

^a^Significant at the 0.05 level

^b^Significant at the 0.01 level

**Table 4:** SMAP results for the TOD corrected embedding

ID	dim	n	nrmse
1	1	107	1.007
2	1	114	1.064
3	1	112	0.872
4	2	111	1.052
5	1	119	1.001
6	1	112	1.055
7	2	106	0.983
8	1	111	0.826
9	1	116	0.913
10	1	110	0.981

Note: ID = subject ID, dim = dimension of the embedding, n = number of vectors in the embedding, nrmse = value of the NRMSE (at θ = 0, the global linear case, local linear models were not applied)

**Table 5:** Intra-embedding distribution of coefficient 1

	Global models								Optimal models					
	Full emb.		Night		Day		TOD		Full emb.		Night		Day	
ID	val	sd	val	sd	val	sd	val	sd	val	sd	val	sd	val	sd
1	− 0.98	0.008	− 0.77	0.014	− 2.25	0.071	−0.95	0.015	− 1.06	0.044	−0.84	0.040	−2.32	0.099
2	−1.02	0.020	−0.82	0.022	−2.11	0.101	− 1.03	0.022	−1.02	0.020	−0.82	0.022	−2.09	0.110
3	−0.98	0.003	−0.93	0.006	−1.06	0.347	−0.95	0.009	−1.11	0.104	−0.94	0.008	−1.06	0.347
4	−1.00	0.010	−0.68	0.013	−2.70	0.105	−1.18	0.029	−1.14	0.072	−0.69	0.017	−2.70	0.105
5	−1.00	0.004	−1.02	0.007	−0.45	0.818	−0.98	0.011	−1.19	0.126	−1.03	0.009	−0.45	0.818
6	−1.00	0.005	−0.90	0.020	−2.22	0.431	−1.08	0.017	−1.16	0.081	−0.90	0.020	−2.27	0.297
7	−1.84	0.035	−0.84	0.013	−3.43	0.156	−1.42	0.028	−2.15	0.649	−1.03	0.119	−3.64	0.308
8	−1.01	0.006	−0.81	0.011	−2.29	0.066	−1.02	0.009	−1.05	0.036	−0.84	0.020	−2.31	0.081
9	−0.99	0.006	−0.94	0.017	−2.35	0.084	−1.04	0.012	−1.12	0.073	−1.33	0.281	−2.50	0.218
10	−0.99	0.004	−0.87	0.013	−1.77	0.620	−0.98	0.014	−1.08	0.056	−0.94	0.049	−1.77	0.620

Note: ID = subject ID, val = average value of coefficient 1 per target, sd = standard deviation of coefficient 1 within an embedding, Night = night bundle, Day = day bundle, TOD = TOD-corrected model

**Table 6:** Bootstrap distribution of coefficient 1 for the optimal models

	Full embedding		Night bundle		Day bundle		TOD corrected	
ID	val	rmsd	val	rmsd	val	rmsd	val	rmsd
1	−1.06	0.10	−0.84	0.14	− 2.20	0.32	−1.18	0.23
2	−1.02	0.22	−0.82	0.16	−2.05	0.37	−1.38	0.34
3	−1.11	0.12	−0.94	0.05	−1.06	0.41	−1.28	0.23
4	−1.14	0.13	−0.69	0.10	−2.31	0.39	−1.28	0.25
5	−1.18	0.17	−1.03	0.05	−0.41	0.81	−1.13	0.17
6	−1.16	0.10	−0.90	0.15	−1.94	0.50	−1.32	0.28
7	−1.95	0.20	−1.03	0.14	−2.74	0.19	−1.74	0.30
8	−1.05	0.07	−0.84	0.09	−2.29	0.25	−1.33	0.18
9	−1.12	0.10	−1.29	0.31	−2.37	0.29	−1.54	0.31
10	−1.08	0.06	−0.94	0.10	−1.54	0.59	−1.60	0.39

Note: ID = subject ID, val = average value of coefficient 1, rmsd = root mean square deviation of the coefficient values relative to the average of the corresponding target

### Full embedding

In nine out of ten participants, the optimal embedding, according to the NRMSE, had a dimension of 1 (Table 2). In eight out of ten subjects, local models performed better than global models, as shown by a θ greater than 0 and a significant difference between the NRMSE bootstrap distribution of the global model (θ = 0) and the NRMSE bootstrap distribution of the optimal model. The average NRMSE of all participants was 0.569.

An examination of the overall distribution of coefficient 1 in the optimal models (Fig. 3) showed sharp distributions in all participants, except for participant 7, whose first coefficient showed a wide bimodal shape. The within-embedding distributions of coefficient 1 in the global models showed sharp peaks for all participants, as was expected (Fig. 4, Table 5). In the optimal embeddings (Fig. 5, Table 5), these distributions were wider, indicating differences between the coefficient values per target within the embedding, which is in line with the expected outcome in the case of the presence of local behavior. Furthermore, most of these optimal distributions showed bimodal features, most probably due to the presence of day and night vectors in the same embedding. Interestingly, the width of the bootstrap distribution of the first coefficient in the optimal models (Table 6) was wider than the width of the intra-embedding distribution (Table 5), except for participant 7, who showed a wider intra-embedding distribution. Therefore, the wide overall shape for this participant may largely be attributed to the intra-embedding variation of the coefficient, while for the other participants, the shape of the overall distribution may mainly be determined by the bootstrap variation.

### Bundle embedding

The night bundles showed an optimal NRMSE at dimension 1, in seven out of ten participants, and at dimension 2 in three out of ten participants (Table 3). The average NRMSE of all participants was 0.671. Only four out of ten participants showed a significant difference between the NRMSE of the global model (θ = 0) and the NRMSE of the optimal model (θ > = 0). An examination of the overall distribution of coefficient 1 in the optimal models (Fig. 3) showed sharp distributions for all participants except participant 7 and 9. The within-embedding distributions of coefficient 1 in the global models showed sharp peaks for all participants, again as expected (Fig. 4, Table 5). In the optimal embeddings (Fig. 5, Table 5), these distributions were wide for participant 7 and 9. Similar to the full embeddings, for these two participants, the width of the overall distributions seems to be determined mainly by the width of the within-embedding variation of the coefficient, while for the other participants, the width may mainly be determined by the bootstrap variation.

The dimensions of the day bundles showed more diversity, with three participants having an optimal NRMSE result at dimension 1, two participants at dimension 2, four participants at dimension 3, and one participant at dimension 7. The average NRMSE of all participants was 1.49. Only three out of ten participants showed a significant difference between the NRMSE of the global model (θ = 0) and the NRMSE of the optimal model (θ > = 0). In general, the overall distributions of all coefficients were wide for all participants (Fig. 3). Furthermore, even in the global case, the within-embedding distribution of coefficient 1 was wide (Fig. 4, Table 5). This may be indicative of the presence of a large amount of noise. In the day bundles, the width of the bootstrap distribution of the first coefficient in the optimal models (Table 6) was similar to the width of the within-embedding distribution (Table 5) for participants 3, 5, 9 and 10. In the night bundles, the bootstrap distribution is wider than the within-embedding distribution for all participants except for participant 5 and 10. This may also be indicative of a large amount of uncertainty.

### Time-of-day corrected embedding

The TOD-corrected embeddings showed an optimal NRMSE at dimension 1 in eight out of ten participants (Table 4). The other two participants showed an optimal NRMSE at dimension 2. The average NRMSE was 0.975. To optimally reflect the common TOD-corrected (linear) method of analyzing time-series data, this analysis was carried out using only a global estimator (θ = 0). In all participants, the within-embedding distribution of coefficient 1 (Table 5) was considerably smaller than the bootstrap distribution (Table 6), indicating that the width of the overall distribution of this coefficient (Fig. 3) was mainly determined by the presence of noise.

### Comparison of embeddings

A comparison of the NRMSE values (Tables 2, 3 and 4) indicated the best prediction performance for the bundled night embeddings, followed by the full embeddings, the TOD-corrected embeddings and the day embeddings. Although the NRMSE indicated a better than chance performance for the full embeddings (average NRMSE = 0.569), it is important to note that it contains no information about the individual NRMSE’s of the morning and night values because the NRMSE for the predictions are based upon a division of the RMSE by the SD of the complete time series. Indeed, when inspected separately, the average NRMSE of the night bundles (0.671) indicated a better than chance prediction whereas the average NRMSE of the day bundles (1.49) indicated a worse than chance prediction. These NRMSE’s are based upon a division of the RMSE by the SD of the night and day observations respectively. The average NRMSE for the TOD-corrected embeddings (0.975) indicated a prediction performance that was only slightly better than chance. This NRMSE was based upon a division by the SD of the TOD-corrected time series.

The average widths of the intra-embedding coefficient distributions, as represented by the SD, were 0.13 for the full embedding, 0.05 for the night bundles, 0.30 for the day bundles, and 0.02 for the TOD-corrected models (Table 5). In the case of the TOD-corrected models this small width was expected, since it is based on a global linear approximation. The smaller intra-embeding width of the night bundles, when compared to the full embeddings, indicated a smaller proportion of local behavior in the night bundles. This was also supported by the smaller average value of θ, having a value 0.26 for the night bundles and 0.50 for the full embeddings (Tables 2 and 3). The intra-embedding coefficient widths of the day bundles were wider than the width in the full embeddings. However, a smaller width was expected because coefficients in a bundle should be more similar to each other than coefficients in a full embedding. Interestingly, the bootstrap distributions of the coefficients in the day bundle were also wider than the bootstrap distributions in the full embedding.

The average widths of the bootstrap distributions were 0.13 for the full embeddings, 0.13 for the night bundles, 0.41 for the day bundles, and 0.27 for the TOD-corrected models (Table 6). This indicated that the uncertainty about the coefficient values was the largest in the day bundles.

## Discussion

This study aimed to evaluate the usefulness of a combination of SMAP and bundle embeddings in the analysis of urinary cortisol time-series data. Comparison of the NRMSEs of unbundled, bundled and TOD-adjusted embeddings showed that the embeddings for the night bundle best predicted future values of cortisol in the time series, followed by the full embeddings. The TOD-corrected embeddings performed only slightly better than chance and the embeddings of the day bundle performed worse than chance. Inspection of the coefficients of the fitted linear models showed that the coefficient distributions of the full embeddings best resembled those of the night bundle embeddings, and it showed that the number of dimensions needed to predict the night values was less than the number needed to predict the day values. Furthermore, in the bundled embeddings, the best results were obtained by using almost global linear models. The full embeddings showed the best results when local linear models were used. Several interesting aspects of these results are discussed in more detail below.

To explain the results, it is important to compare the variations of the non-differenced night values and the non-differenced day values (Table 1). The day values show more variation than the night values, making it easier to predict the first-differenced night values than the first-differenced day values. When fitting the linear models, CIs for coefficients of models that are used to predict the differenced night values will be narrower than the CIs of models for the differenced day values. As a consequence, the coefficients for the linear models that are fitted to the full embeddings may predominantly resemble the linear models for the night bundles because the coefficients of these night models have only little freedom of variation during the fitting procedure. This comparatively better performance of the night models may also explain why the dimensions of the models for the full embeddings are mostly similar to the dimensions of the models for the night bundles (see Tables 2 and 3).

From a psychophysiological perspective, the lower variance in the night values in comparison with the day values may reflect the relative absence of external influences during the night as well as the absence of the influence of events that have occurred earlier in time. Indeed it has been shown that cortisol levels can peak quickly in response to psychosocial stressors [14], which could explain the relatively high variance in cortisol levels during the day, when exposure to (multiple) psychosocial stressors of differing intensity is most likely. Consequently, the night values may be better suited for the investigation of long-term changes in the cortisol system, although the values of the dimensions did not seem to support the presence of such long-term processes in the current data.

Inspection of the values of θ of the optimal Gaussian weight curve showed better prediction performance for the local predictors in the case of full embeddings, which is also reflected by significant differences between the prediction accuracy of the global and local models in 8 out of 10 embeddings. In the night and day bundles such superior performance of local prediction was less evident. Possibly, the local behavior in the full embeddings is mainly caused by the underlying diurnal pattern, leading to better prediction with different local-linear parameters for the night and day values. Once these values are separated by means of the night and day bundles, the necessity for local parameters may be largely eliminated.

The absence of local behavior after the full embedding is separated into bundles seems to indicate the absence of nonlinear dynamical contributions on the timescale of days. Although the cortisol system is expected to show nonlinear behavior, it may be that such nonlinearity is only measurable on a timescale of minutes to hours, making it impossible to capture it with only two measurements per day. In this light, it is interesting that cortisol is known to show ultradian rhythms that consist of one or more cortisol pulses within a time window of several hours and occur up and above the regular diurnal pattern [15]. Another possibility is that long-term nonlinear contributions are obscured by linear stochastic contributions, measurement error and noise.

The current study used a nonlinear predictor (SMAP) to find the dimensions of the optimal embeddings. However, the results showed that most of the local behavior disappeared when bundle embeddings were used. This may imply that the current time series could have been analyzed equally well with regular time-series methods, provided that these series would have been split in different sets for different TODs in the same way as used for the current bundling approach. That is, separate linear models would have to be fitted for the night values and the day values (whereby the night-value models would still use day values in the predictor vector, and vice versa). However, when there are influences that have the same linear contributions in both TODs, it may be that splitting up the data in this way would take away the possibility to find these influences because of a decrease of power due to the lower number of data points per set.

Strengths of the current study are (1) the use of SMAP, which allows to fit global as well as local linear models, and thus can capture any present nonlinear influences; and (2) the use of bundle embeddings, which allow for the use of nonlinear analysis methods in the presence of coupled periodically varying external variables (i.e. TOD). However, the study also has some weaknesses. First, the current study may have been limited by the fact that the time series had a length of only 126 measurements and measurements were conducted only twice per day. It may be that a higher number of measurements per day would have revealed the presence of intraday nonlinear behavior. Second, urinary cortisol is a measure of accumulated cortisol during an interval. Analysis of more instantaneous measurements (e.g. blood cortisol) could yield different results. Third, although the power of the analysis depends on the number of measurements per person, due to the low number of participants, generalizability of the results to the population at large may be limited. Finally, the used analytical strategy may also have limitations. For instance, using bundle embeddings may lead to a decrease of power in the presence of similar linear contributions for each TOD. In addition, the use of bootstrapping to estimate CIs for full and bundle embeddings can make the analyses time-consuming. Considering the abovementioned issues, suggestions for further research include the use of longer time series, with a higher number of measurements per day, and the use of linear time-series models on datasets that are split according to the bundling approach.

## Conclusions

In conclusion, the current study showed that using a bundling approach on time series of cortisol may reveal differences between the predictions of night and day cortisol that are difficult to find with conventional time-series methods. Combination of this approach with SMAP may especially be of use when analyzing time-series data that contain periodic components, possibly due to coupling with an external variable.

## Abbreviations

AR: Autoregressive
CI: Confidence interval
NRMSE: Normalized root mean square error
RMSE: Root mean square error
SD: Standard deviation
SMAP: Sequentially weighted global linear map
TLS: Total least squares
TOD: Time of day
